# Effects of quince seed, almond, and tragacanth gum coating on the banana slices properties during the process of hot air drying

**DOI:** 10.1002/fsn3.489

**Published:** 2017-07-06

**Authors:** Reza Farahmandfar, Maedeh Mohseni, Maryam Asnaashari

**Affiliations:** ^1^ Department of Food Science and Technology Sari Agricultural Sciences & Natural Resources University (SANRU) Sari Iran; ^2^ Department of Food Science and Technology Khazar Institute of Higher Education Mahmoudabad Iran

**Keywords:** Almond gum, Banana slices, Drying, Quince seed gum, Tragacanth gum

## Abstract

Due to early deterioration of banana in drying process, almond, quince seed, and tragacanth gums as edible coatings were determined. For this purpose, banana slices were coated in 0.7% solution of each gum and one group remained uncoated as the control. The samples were examined at specific times considering the weight loss, color analyzing (a*, b*, and L*) through the method computer vision, color difference index, browning index, and rehydration after the samples being dried. The results showed that the weight loss of the coated samples was significantly (*p* < .05) higher than the uncoated samples which can be due to the alteration or destruction of the cell membrane. The almond gum‐coated samples had significantly a lower ultimate browning index and quince seed gum‐coated samples showed the highest rehydration. So, the gums coating is an effective way to preserve the quality characteristics of the banana slices.

## 1 INTRODUCTION

Banana is a popular, delicious, and nutritious fruit that grows in tropical, subtropical, and humid regions. Banana plants are from large herbaceous perennial plants similar to shrubs, but it has grass stems, large leaves that tent at the end of its foliar trunk whose inflorescence appears between the leaves. Banana is introduced as a beneficial food because of its high energetic value. High potassium in banana causes an anticancer effect. Moreover, banana is effective in the treatment of allergies, hives, and itchy skin, relieving cough and hoarseness, wound healing and preventing it from bleeding, curing dermal black spots, reinforcing the stomach and sexual power, and developing and balancing the nervous system (Hailu, Workneh, & Belew, 2013; Singh, Singh, Kaur, & Singh, 2016). However, after harvesting and slicing, texture injuries, such as browning, dehydration, and product shrinkage, increasing microbial activities, and also softening in the slices tissues were accelerated (Chauhan, Raju, Singh, & Bawa, 2011). Therefore, it is necessary for the banana slices to be processed in order to extend its shelf life.

Drying fresh slices of perishable fruits and vegetables improves the stability of the final product by reducing the moisture content and water activity, microbial activities, and the speed of chemical reactions and inactivating enzymes (Asnaashari, Tajik, & Khodaparast, 2015). Reduce the weight and volume of product, storage and packaging costs, and facility of transportation are all other advantages of drying (Dhall, 2013; Doymaz & İsmail, 2011). Hot air drying is the most common method of drying fruit slices. In this method, the heat is transferred to the product by moving the hot air which makes the moisture come from the inside to the surface of the product and evaporate. Therefore, the heat transfer and mass transfer both occur simultaneously (Thuwapanichayanan, Prachayawarakorn, Kunwisawa, & Soponronnarit, 2011).

For drying fruits contain too sugars—like bananas—using hot air flow, higher deals of temperature and time are required which can cause serious injuries to the dried product (Maskan, 2000). On the other hand, deploying particular techniques is necessary to prevent oxidation and enzymatic browning reactions and then to preserve the product quality characteristics such as tissue, color, and aroma (Baini & Langrish, 2008). Using edible coatings is one of these techniques that make the optimized quality and shelf life become achieved by performing the minimal processes on the foodstuff.

Gums are polysaccharide polymers which are used as edible coatings in various foods (Asnaashari, Motamedzadegan, Farahmandfar, & Rad, 2016). They can be used as emulsifier, stabilizer, thickener, inhibitor of sugar and ice crystals formation due to their hydrocolloid properties (Farahmandfar, Asnaashari, Salahi, & Rad, 2017; Weiping & Branwell, 2000). In recent years, scientists are looking for newer source of gums with their excellent antioxidant properties such as *Amygdalus scopsria zedo* gum as an arabinogalactan gum (Fadavi, Mohammadifar, Zargarran, Mortazavian, & Komeili, 2014).

The quince fruit (*Cydonia oblonga*) is a native fruit of West Asia region which is often processed (e.g., jam) to be consumed. Pharmaceutical benefits are also mentioned for quince to treat dysentery, ulcers, cancer, and sore throat and its mucilage is used for encapsulation of essential oils (Abbastabar, Azizi, Adnani, & Abbasi, 2015; Ritzoulis et al., 2014). The quince seed contains phenolic compounds and acids, such as: citric acid, malic acid, fumaric acid, ascorbic acid, quinic acid, and shikimic acid, and also amino acid, such as glutamic acid, aspartic acid, and asparagine (Silva et al., 2005). The investigations have showed that arabinose, xylose, galactose, and glucose are obtained from quince seed gum hydrolysis (Vignon & Gey, 1998). Quince seed gum has good emulsifying properties and is a good carrier for antioxidant and antimicrobial compounds.

Almond gum is a gum that seeps from the almond tree which contains 92.36% polysaccharides and sugars including: arabinose, galactose, and uronic acid. Therefore, they are considered among the arabinogalactan polymers. The amount of protein and lipids in almond gum is low, but this gum contains mineral components including calcium, magnesium, sodium, and zinc (Bouaziz et al., 2015). Almond gum has almost the same emulsifying properties as Arabic gum. However, at higher concentration, the emulsifier effect of almond is higher (Mahfoudhi et al., 2014). Besides, the oligosaccharides of almond gum can be used as a natural preservative due to good antioxidant potential to prevent lipid oxidation and a high antimicrobial activity versus the bacteria and molds (Bouaziz et al., 2015).

For many years, the tragacanth gum is used as an efficient hydrocolloid in the food, pharmaceutical, cosmetics, textiles, and leather industries (Balaghi, Mohammadifar, Zargaraan, Gavlighi, & Mohammadi, 2011). Tragacanth is hydrophilic, heterogeneous, and highly branched hydrocolloid (Chenlo, Moreira, & Silva, 2010). Tragacanth gum consists of two components: 1. Bassorin (insoluble in water and capable of swelling and forming gel) and 2. Tragacanthin (soluble in water and capable of the forming colloidal solution); and the following sugar: arabinose, xylose, glucose, fructose, galactose, rhamnose, and galacturonic acid (Balaghi, Mohammadifar, & Zargaraan, 2010; Weiping & Branwell, 2000). This gum has good emulsifying properties, suspension performance, and stability to heat and acidity which is widely used in various foods as a stabilizer, thickener, and lipid alternative (Balaghi et al., 2010). Mohebbi, Ansarifar, Hasanpour, and Amiryousefi (2012) examined the effect of tragacanth gum and aloevera gel on the physicochemical properties of bell pepper at different storage temperatures and observed that the coatings had better effects which postponed the color changes, softening, and shrinkage. Tavakolipour and Zirijany (2014) coated the banana slices with citric acid and ascorbic acid after blanching. This action reduced the samples drying time up to 52% compared to the uncoated samples during the drying by hot air and microwave. Askari, Emam‐Djomeh, and Mousavi (2008) coated the apple slices with the starch, pectin, carboxymethyl cellulose, and calcium chloride after blanching. Then, they dried the samples under the hot air and microwave. They reported that this action would increase the porosity greatly and obtain a product with a massive and porous tissue. Thuwapanichayanan et al. (2011) also immersed the banana slices in ascorbic acid and then dried at 70–100°C. The results showed that high temperature (70–90°C) had no significant effect on browning, however, the loss of total volatiles at higher temperatures was less than the others.

In this investigation, the computer vision method was deployed to analyze the color changes. This method is the digital, computer‐analyzed images which give us useful data to control the processes without any unfavorable effects on the products. So, the aim of this study is to evaluate the effect of quince seed, almond, and tragacanth gums on the features of hot‐dried banana slices which is performed for the first time.

## 2 MATERIALS AND METHODS

### 2.1 Materials

To conduct this research, dried quince seeds and strip‐shaped tragacanth gum were purchased from local market in Isfahan. Almond gum also was provided from Forests, Range and Watershed Management Organization in Isfahan. Sumifru Gracio Philippines Bananas with the same size, smooth, good‐looking yellow, and free of corrupting or nigrescence spots were purchased from local stores and were stored at 13°C until the testing time.

### 2.2 Preparing the samples

Fruit skins were washed under water, then became peeled off and sliced to the thickness of 5 mm using a sharp stainless steel knife on a flat surface. Then, a certain number of the slices were randomly used as the control samples or as sample for the three gum coating.

### 2.3 Preparing the quince seed gum solution

The quince seed gum was prepared based on the method suggested by Jouki, Yazdi, Mortazavi, and Koocheki (2014) according to the following instruction: After the seeds sorting was done, 50 g of them was weighted, and then, the distilled water (1,250 ml) was poured. This mixture was left steadily for 2 hrs at the room temperature and after elapsing this time, the mixture was passed through the filter and was centrifuged in a refrigerated centrifuge (Centurion Scientific Model: K3 series; Centurion Co., UK) at 6,000 rpm 4032 g for 5 min. In the next stage, centrifuged solutions were passed through a filter and liquid gum was obtained. The prepared liquid was moved into the oven and left to be dried for about 16 hrs at 50°C. This dried gum turned into powder and became sieved. Ultimately, 0.7 g of the powdered quince seed gum was weighted and slowly stirred at 40°C, gradually 100 ml distilled water was added to become well dissolved in the distilled water.

### 2.4 Preparing almond gum solution

The dried almond gums turned into powder deploying an electric mill. The resulting powder was left in the distilled water for one night, and then, it was sieved to remove the impurities. 0.7 g of this gum was weighed using a digital scale (Multi‐function Electronic Scale, MH‐200, china) and gradually added to 100 ml of distilled water at 40°C. Stirring the solution was done to obtain a complete dissolving of the gum.

### 2.5 Preparing the solution of tragacanth gum

Strip‐shaped slices of tragacanth turned into powder using an electric mill. Powdered samples also were sieved in order to obtain the powder particles in the same sizes. After that, 0.7 g of the gum powder was separated and such as the methods were mentioned for the almond gum, a 0.7% tragacanth gum solution was prepared.

### 2.6 Coating the banana slices

After preparing the 0.7% solutions from each gum, the solutions were left for 1 hr at room temperature for water absorbing of gums becomes well done. The banana slices were divided into four groups with a certain number. After being sliced, three groups of them were instantly immersed in the gum solution one by one for 2 min. Then, the slices were ejected and left steadily for a few minutes until the gums penetrate into the internal layers of the fruit tissues and the additional gums also become ejected. The sliced samples were instantly transferred to dryer after experiment, while the last group, namely, the group of the control samples also after being sliced and going under the initial experiments was moved to the dryer without any kind of coatings.

### 2.7 Drying

The coated and uncoated banana slices were placed on the perforated tray at similar distances to the center of the dryer. The tray was moved into the dryer chamber (Delmonti: DL190, China). The hot air drying was performed at the temperature 135°F (58°C) at the specific interval: 30, 60, 90, 150, 210, and 270 min after starting the process, the samples were ejected from the drier and went under the experiments.

### 2.8 Weight loss analyzing

At the specific intervals, the samples were ejected from the dryer and were weighed deploying a digital scale. The reported samples weight loss was calculated based on percentage of initial weight according to the following formula (1):

(1) $$Weightloss\%=\left(\frac{initial\;weight-Dried\;sample\;weight}{initial\;weight}\right)\times 100$$

### 2.9 Shrinkage analyzing

Change in size and diameter toward smaller defined as shrinkage. Reason of this change was egress of moisture of banana slices during storage, which cause change pressure balance between inside and outside banana. This stress leads shrinkage in material. Apparent shrinkage (Sapp) is defined as the ratio of the apparent volume at a given moisture content (*V*_app_, m³) to the initial apparent volume of materials before processing (*V*_0app_, m³) (Sahin & Sumnu, 2006):

(2) $$S_{\mathrm{app}}=\frac{V_{\mathrm{app}}}{V_{0\mathrm{app}}}\times 100$$

### 2.10 Measuring the color

Each of the samples was scanned at the determined times deploying the scanner (HP Scanjet: 3,770) with a resolution of 3,600 saved in PNG format. In order to eliminate the additional lights and to neutralize their effect on the image scanning, a carton box was placed on the scanner. The images were analyzed under the application ImageJ 1.46r. For this purpose, an identical layer of the whole images was selected at first. Then, the amounts of the color parameters: L*, a*, and b* were determined using the plug‐in of the color space converter. Positive and negative values of the parameter b* are represented in yellow and blue, whereas positive and negative values of the parameter a* are represented in red and green (Eshghi, Asnaashari, Haddad Khodaparast, & Hosseini, 2014). The L* parameter also represents the lightness whose value ranges between 0 (for black) and 100 (for the complete reflection of light) (Sun, 2016).

### 2.11 Color difference index (ΔE) and browning index (BI)

Color changes during the drying process are calculated through the ΔE index (color difference in the sample compared to the prototype) according to the following formula. The more color changes during the process, the higher amount of this index:

(3) $$\Delta E=\sqrt{{\left(L^{*}-L_{0}^{*}\right)}^{2}+{\left(a^{*}-a_{0}^{*}\right)}^{2}+{\left(b^{*}-b_{0}^{*}\right)}^{2}}$$

Browning index (BI) is one of the most important reported parameters in the both enzymatic and nonenzymatic browning processes. This index indicates the brown color purity which can be calculated in this way (Perez‐Gago, Serra, & Del Rio, 2006):

(4) $$\text{BI}=\left(\frac{100\;\times \;\left(x\;-\;0.31\right)}{0.172}\right)$$

(5) $$x=\frac{a^{*}+1.75L^{*}}{5.645L^{*}+a^{*}-3.012b^{*}}$$

### 2.12 Measuring the rehydration

Measuring the rehydration of the dried banana sample slices was performed based on the method suggested by Tavakolipour and Zirijany (2014). To do this, after 270 min of the drying process, the samples were weighed initially and then they were immersed in a beaker containing distilled water at room temperature for 15 hrs. This time is the period which no more weight gain is observed. After this time, the samples were ejected from the distilled water and the surface water was taken away and put on a filter paper (Whatman 1). The samples were weighted and their reabsorption capacity was calculated in the following way:

(6) $$\text{RC}=\frac{W_{r}}{W_{d}}$$

(RC: rehydration capacity of the sample; *W*_r_: ultimate weight after rehydration; and *W*_d_: dry sample weight)

## 3 STATISTICAL ANALYSIS

All tests were performed in triplicate. To investigate the effects of gum on banana slices during the drying process, the analysis of variance (ANOVA) was used in SPSS.19 software. Duncan test at the significance level (*p* < .05) was performed. The data figure was drawn in Excel software (2010) based on the average of three replications.

## 4 RESULTS AND DISCUSSION

### 4.1 Weight loss and shrinkage of banana slices

All of the samples almost lost 24.36 ± 2.55% of their weight during the first 30 min of hot air drying process which indicates their quick water loss. As time passed, the slop of weight loss trend was sharper, but the amount of weight loss in all of the coated slices was significantly (*p* < .05) higher than the uncoated ones (Table 1).

**Table 1:** The amounts of weight loss in the coated and uncoated banana slices during the drying process

Treatments	Time (mins)					
	30	60	90	150	210	270
Uncoating	25.07 ± 1.57^a^	38.48 ± .63^a^	46.94 ± .99^a^	60.64 ± 1.17^a^	68.51 ± .26^a^	72.11 ± .12^a^
Almond gum coating	23.36 ± 3.68^a^	38.67 ± 4.99^a,b^	50.13 ± 5.32^a,b^	64.04 ± 6.10^a,b^	72.35 ± 4.53^a,b^	76.20 ± 2.31^b^
Tragacanth gum coating	24.96 ± 2.35^a^	44.30 ± 2.37^b^	54.01 ± 2.86^b^	68.21 ± 3.39^b^	76.44 ± .74^b^	78.62 ± .62^b^
Quince seed gum coating	24.07 ± 2.61^a^	39.17 ± 1.42^a,b^	49.95 ± .23^a,b^	66.26 ± .96^a,b^	75.43 ± .80^b^	78.45 ± .91^b^

Different letters in each column show significant difference at *p* < .05.

During the hot air drying process, the moisture is initially removed from the slice surface and then the interior moisture comes to the surface by moisture diffusion mechanism and finally, the moisture evaporates. When the fruit slices are exposed to hot air for some time, the rate of water loss and, consequently, the amount of weight loss reduce gradually because the moisture content is reduced. However, temperature still hurts the cell membranes due to an internal pressure and then reduces the moisture distance from interior part of slices to reach the surface. On the other hand, any alteration that occurs in the cell membrane can enlarge the surface for the moisture to be released (Thuwapanichayanan et al., 2011). Gum coating may also enlarge the surface to make the product water released. After water evaporation of the coated samples from the surface, the gum particles could absorb water from the interior parts because of their hydrocolloid properties which accelerate the moisture transfer to the surface. Besides, due to the polysaccharide features of the gums, they may react with some components of the banana slices causing the moisture to be released more easily and the water to be released more conveniently and also a further reduction in weight. The relative humidity of interior part of fruit is high which causes a higher amount of vapor permeability due to the coating process (Perez‐Gago, Serra, Alonso, Mateos, & Del Río, 2005). The higher weight loss in a coated sample indicates that protein or polysaccharide coatings alone do not provide a good barrier to moisture (Krochta, 1996), but it has the advantage that the substance will be exposed to hot air for a shorter time period to reach the desired moisture and therefore the tissue and product quality will become less damaged. Lenart and Piotrowski (2001) also mentioned that coated fruits with starch and pectin solutions showed a higher loss ratio of water compared to uncoated ones. Among the gum‐coated samples, almond gum coating led to a lower weight loss amount compared to quince and tragacanth gum coating which is perhaps due to containing small amounts of lipids and minerals like calcium or sodium.

The coated samples showed less shrinkage compared to uncoated ones. Indeed, creating porosity in the dried fruit tissue also can be a reason for a less shrinkage in the coated samples (data not shown).

### 4.2 Color of banana slices

The color can be an indicator of microbial and enzymatic activity or quality of products during the drying process. The most important factor for discolorations of sliced fruits is the browning due to enzymatic activities, in particular, the polyphenol oxidase enzymes. Phenolic compounds are the main substrates of these enzymes activities.

The banana slices were initially yellowish white (milky), but their colors tended into yellow brown after the hot air flow drying process. The changes in the amount of color parameters a*, b*, and L* during the drying process can be seen in Table 2. In the first few minutes of the drying process, the increasing trends of a* and b* were high, but during the process time, a more moderate increasing trend was observed. The amounts of a* and b* were little initially in the uncoated samples compared to the other samples, but ultimately, the amounts of these two color parameters were higher than what of the coated samples which indicates that the process is critically effective on these samples. The greatest amount of initial L* was observed in the almond gum‐coated samples, whereas the lowest amount was observed in the quince seed gum‐coated samples. Between the basic solutions prepared from the three gums for coating the slices, the most transparent solution was the solution obtained from the almond gum while the solution made of quince seed gum was opaque (light brown) which can be the cause of difference between the initial L* rates in the samples. The amount of L* was reduced in all of the banana slice samples during the hot air drying. The ultimate amounts of L* of the uncoated samples were significantly lower (*p* < .05) than L* of the coated samples and L* of almond and tragacanth gum coatings samples were greater than the other samples.

**Table 2:** The changes in the color parameters (a*, b*, and L*) in the banana slices during the hot air drying

Treatments	Time (mins)						
	0	30	60	90	150	210	270
b*							
Uncoating	24.84 ± 0.48^a^	32.61 ± 0.39^b^	34.20 ± 0.38^b^	34.73 ± 0.71^b^	35.51 ± 0.60^b^	35.77 ± 0.63^b^	36.25 ± 0.31^b^
Almond gum coating	24.52 ± 0.28^a^	30.00 ± 0.55^a^	32.38 ± 1.34^a^	32.54 ± 1.88^a^	32.62 ± 0.71^a^	33.05 ± 0.54^a^	33.20 ± 1.23^a^
Tragacanth gum coating	25.33 ± 1.02^a^	29.23 ± 0.92^a^	31.80 ± 0.51^a^	32.50 ± 0.38^a^	33.61 ± 0.67^a^	34.50 ± 0.88^a,b^	35.46 ± 0.12^b^
Quince seed gum coating	27.01 ± 1.09^b^	32.19 ± 0.39^b^	33.30 ± 0.58^a,b^	33.52 ± 0.23^a,b^	33.91 ± 1.09^a^	34.59 ± 1.19^a,b^	34.82 ± 1.38^a,b^
a*							
Uncoating	−1.95 ± 0.34^a^	1.19 ± 0.03^b^	1.40 ± 0.08^b^	2.28 ± 0.14^c^	3.13 ± 0.57^b^	3.43 ± 0.59^a^	4.45 ± 0.55^a^
Almond gum coating	−1.70 ± 0.14^a^	0.71 ± 0.31^a,b^	1.12 ± 0.20^a,b^	1.34 ± 0.21^a,b^	2.02 ± 0.15^a,b^	2.36 ± 0.46^a^	3.41 ± 0.03^a^
Tragacanth gum coating	−1.88 ± 0.13^a^	0.28 ± 0.45^a^	0.81 ± 0.49^a^	0.92 ± 0.52^a^	1.78 ± 1.02^a^	2.43 ± 1.44^a^	3.36 ± 0.89^a^
Quince Seed gum coating	−1.40 ± 0.99^a^	0.99 ± 0.61^a,b^	1.46 ± 0.24^b^	1.54 ± 0.11^b^	2.13 ± 0.18^a,b^	3.40 ± 0.73^a^	4.39 ± 0.58^a^
L*							
Uncoating	84.40 ± 0.57^a^	74.28 ± 0.79^a^	69.89 ± 0.13^a^	67.13 ± 0.20^a^	65.56 ± 1.82^a^	65.29 ± 0.17^a^	64.75 ± 0.19^a^
Almond gum coating	85.02 ± 0.85^a^	76.10 ± 0.79^a^	73.37 ± 0.89^b^	71.83 ± 0.59^b^	70.86 ± 1.13^b^	70.39 ± 0.30^c^	69.94 ± 0.84^c^
Tragacanth gum coating	83.81 ± 0.63^a^	76.53 ± 1.55^a^	72.82 ± 2.09^b^	70.96 ± 2.58^b^	70.80 ± 1.75^b^	69.95 ± 1.34^c^	69.42 ± 1.12^c^
Quince seed gum coating	82.66 ± 3.07^a^	75.34 ± 1.67^a^	71.78 ± 1.66^a,b^	69.29 ± 0.87^a,b^	68.98 ± 0.92^b^	68.13 ± 0.32^b^	67.53 ± 0.54^b^

Different letters in each column show significant difference at *p* < .05.

The most of the L* changes in the product color variations caused by the activity of enzymes. Stress (coating or drying in this research) can lead to release polyphenol oxidase from the cells to react with phenolic compounds and oxygen and make brown compounds (Salvia‐Trujillo, Rojas‐Graü, Soliva‐Fortuny, & Martín‐Belloso, 2015). Although increasing a* and decreasing L* indicate the banana slices browning. But, b* as yellowing index indicates that lighter brown is created through the color combination makes the product to not misrepresent. The slices physical view also supports this idea.

After 90 min of drying, a fairly significant increasing trend was observed in a* amounts of the samples which seems to be as a result of the Millard reaction regarding the denaturation of the most enzymes. Either the temperature rising or longer exposure to the heat would cause an increasing trend in both the pigments production and the intensity of brown color due to the Millard reaction.

Between the coated samples, the amount of a* was found in the quince seed gum‐coated samples more than the others and the L* was significantly lower than the two other gum coatings. Besides the color of the basic gum solution, lightly amount of phenolic compounds of quince seed gum can be used as a substrate of the enzymes.

The temperature of hot air flow not only leads to make the enzymes denatured but it also leads to reduce the water activities in the slices which would rebate the enzyme activities.

### 4.3 Color difference index and the browning index of banana slices

Although the color difference (ΔE) generated did not show any significant differences between the coated samples compared to each other, the ΔE in the uncoated samples was significantly more than the ΔE in the coated samples (Table 3). During fruit slices drying, with hot air flow, a pressure occurs to the fruit structure which increases the color difference and some physical and chemical changes. The color change caused by the operation pressure would indicate the destruction of the samples pigments which intends the rate of browning process (Souza, Medeiros, Magalhães, Rodrigues, & Fernandes, 2007). Dixon and Paiva (1995) mentioned that whenever a group of plant cells are damaged due to the effect of microbial growth and/or any other stress factors, their response mechanism is the phenolic compounds production. Fruit slicing might be one of the stresses that causes the increasing phenolic contents which accelerate phenolase enzymes activities.

**Table 3:** The color difference index (ΔE) in the gum‐coated and uncoated banana slices during the drying process

Treatments	Time (min)					
	0	30	60	90	120	150
Uncoating	13.14 ± .15^b^	17.59 ± .12^b^	20.35 ± .12^b^	22.24 ± .30^b^	22.67 ± .10^b^	23.61 ± .14^b^
Almond gum coating	10.75 ± .14^a,b^	14.34 ± .13^a^	15.73 ± .33^a^	16.73 ± .38^a^	17.42 ± .23^a^	18.14 ± .29^a^
Tragacanth gum coating	8.54 ± .14^a^	13.04 ± .32^a^	14.98 ± .42^a^	15.85 ± .45^a^	17.17 ± .45^a^	18.37 ± .36^a^
Quince seed gum coating	9.28 ± .33^a^	12.89 ± .40^a^	15.16 ± .46^a^	15.72 ± .44^a^	17.07 ± .55^a^	17.98 ± .58^a^

Different letters in each column show significant difference at *p* < .05.

Hot air flow on the product tissue makes release the enzyme:polyphenol oxidase would cause the enzymatic browning of banana slices sample initially. At first, hot air which is flows on the product tissue makes release the enzymes especially polyphenol oxidase. This enzyme would cause the enzymatic browning of banana slices. After that, as the temperature of the hot air increased, the samples denaturation gradually developed. However, temperature of hot air gradually causes the samples denaturation. During the drying process, there is also the possibility of nonenzymatic browning (Millard). If the drying process continues for a long time, it can cause superficial burn spots in the product; all of which affect the discolorations of the material.

During the drying process, the slices browning (BI) increased due to the reasons mentioned. So that, after 270 min, the BI of banana slices was almost doubled and in some cases, more than this level was also reached. The browning rate in the uncoated samples, after 60 min of drying, was significant increased compared to the coated samples (*p* < .05) (Table 4). Between the coated samples, the almond gum‐coated samples indicated the lowest browning rate regarding the low levels of a* and b*, whilst its great L*. The almond gum‐coated samples indicated the lowest browning rate regarding low levels of a* and b* and high level of L* in comparison to other coated samples. On the other hand, the slightness of BI in the samples coated with tragacanth gum compared to the samples coated with quince seed or uncoated samples is probably because of their more abundant methyl groups in Isfahan tragacanth gum rather than the other tragacanth gum species (Gavlighi, Meyer, Zaidel, Mohammadifar, & Mikkelsen, 2013). Indeed, the methylated substrates are not capable of browning by the phenolase enzymes.

**Table 4:** The browning index (BI) changes in the gum‐coated and uncoated banana slices during the drying process

Treatments	Time (min)						
	0	30	60	90	150	210	270
Uncoating	31.78 ± .01^a^	56.42 ± .02^b^	65.32 ± .01^c^	71.47 ± .03^b^	77.12 ± .07^b^	78.71 ± .02^c^	82.33 ± .02^c^
Almond gum coating	31.23 ± .01^a^	48.75 ± .01^a^	56.73 ± .03^a^	58.93 ± .09^a^	60.96 ± .05^a^	62.95 ± .03^a^	65.03 ± .04^a^
Tragacanth gum coating	32.92 ± .02^a,b^	46.42 ± .01^a^	55.66 ± .01^a^	59.34 ± .02^a^	63.16 ± .04^a^	67.17 ± .03^b^	71.44 ± .01^b^
Quince seed gum coating	36.74 ± .05^b^	54.28 ± .02^b^	60.93 ± .04^b^	64.49 ± .01^a^	66.56 ± .06^a^	70.95 ± .05^b^	73.60 ± .04^b^

Different letters in each column show significant difference at *p* < .05.

### 4.4 Rehydration of banana slices

**Figure 1:**
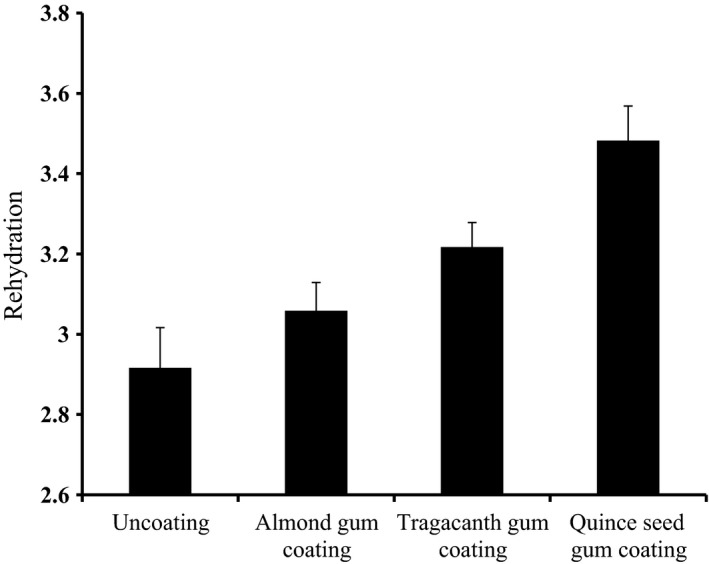
The rehydration amounts in the (coated with almond, quince seed, and tragacanth gums and uncoated) dried banana slices

All samples had lower water absorption rather than the initial weight, but the water absorption was greater in the gum‐coated samples in which the quince seed gum‐coated samples had the most amounts of water absorption (Figure 1). The reason of such a further intensity can be the hydrocolloid nature of the gums and better stability to heat in the coated samples. As banana contains a lot of sugary materials, the more time needed for drying causes more damage to its cell structure. Alteration or disintegration of the cells can inhibit the attaching of water to the previous positions. As a consequence, the rehydration process would be reduced (Damodaran, Parkin, & Fennema, 2007). Lozano, Rotstein, and Urbicain (1980) also have mentioned the cavities blockages due to the cell disintegration as a reason of such a rehydration reduction. Moreover, the temperature causes the loss of hydrogen bonds and deformation of the dried products will reduce the possibility of reestablishing the hydrogen bonds.

The shrinkage due to the drying process would generally reduce the diameter and water absorption amounts in the capillary tubes, but in the gum‐coated samples, the rate of moisture loss was more than the samples without any of the gum coating methods which causes lower shrinkage and facilitate more water absorption in the dried samples (Sacilik & Elicin, 2006).

## 5 CONCLUSION

Nowadays, gums are used extensively in various fields of food industry. This research showed that almond gum, quince seed gum, and tragacanth gum as the coating agent for fruit slices through the drying process. Coating‐dried fruit slices have lower amounts of shrinkage, greater tissue quality, more water loss rates, and also more rehydration, lower browning index, and higher lightness rather than the ones that were dried without any coatings. All these factors indicate that the application of these gum coatings causes better preservation of the dried sliced fruits quality characteristics, saving time, expenses, and the energy consumption in drying process.

## CONFLICT OF INTEREST

None declared.
